# Point-of-care CD4 testing: Differentiated care for the most vulnerable

**DOI:** 10.7189/jogh.12.04004

**Published:** 2022-02-05

**Authors:** Elizabeth Spooner, Tarylee Reddy, Nobuhle Mchunu, Shabashini Reddy, Brodie Daniels, Noluthando Ngomane, Nozipho Luthuli, Photini Kiepiela, Anna Coutsoudis

**Affiliations:** 1 Department of Paediatrics and Child Health, University of KwaZulu-Natal, Durban, South Africa; 2 South African Medical Research Council, HIV Prevention Research Unit, Durban, South Africa; 3 South African Medical Research Council, Biostatistics Unit, Durban, South Africa; 4 South African Medical Research Council, Durban, South Africa; 5 Occupational Health, Durban, South Africa; 6 eThekwini Health Unit, Durban, South Africa; 7 School of Mathematics, Statistics and Computer Science, University of KwaZulu-Natal, Pietermaritzburg, South Africa

## Abstract

**Background:**

South Africa, with the highest burden of HIV infection globally, has made huge strides in its HIV/ART programme, but AIDS deaths have not decreased proportionally to ART uptake. Advanced HIV disease (CD4 < 200 cells/mm^3^) persists, and CD4 count testing is being overlooked since universal test-and-treat was implemented. Point-of-care CD4 testing could address this gap and assure differentiated care to these vulnerable patients with low CD4 counts.

**Methods:**

A time randomised implementation trial was conducted, enrolling 603 HIV positive non-ART, not pregnant patients at a primary health care clinic in Durban, South Africa. Weeks were randomised to either point-of-care CD4 testing (n = 305 patients) or standard-of-care central laboratory CD4 testing (n = 298 patients) to assess the proportion initiating ART at 3 months. Cox regression, with robust standard errors adjusting for clustering by week, were used to assess the relationship between treatment initiation and arm.

**Results:**

Among the 578 (299 point-of-care and 279 standard-of-care) patients eligible for analysis, there was no significant difference in the number of eligible patients initiating ART within 3 months in the point-of-care (73%) and the standard-of-care (68%) groups (*P* = 0.112). The time-to-treat analysis was not significantly different in patients with CD4 counts of 201-500 cells/mm^3^ which could have been due to appointment scheduling to cope with the large burden of cases. However, in patients with advanced HIV disease (CD4 < 200cells/mm^3^) 65% more patients started ART earlier in the point-of-care group (HR 1.65 (95% confidence interval (CI) = 0.99-2.75; *P* = 0.052) compared to the standard-of-care group.

**Conclusions:**

Point-of-care testing decreased time-to-treatment in those with advanced HIV disease. With universal test and treat for HIV, rollout of simple point-of-care CD4 testing would ensure early diagnosis of advanced HIV disease and facilitate differentiated care for these vulnerable patients as per the World Health Organisation 2020 target product profile for point-of-care CD4 testing.

**Trial registration:**

ISRCTN14220457.

South Africa bears more than double the HIV burden and antiretroviral treatment (ART) numbers of any other country [1], as well as 10% of the world’s AIDS related deaths [2]. Huge strides have been made in decreasing incidence, increasing life expectancy [3] and decreasing deaths, but the AIDS death rate has not declined as rapidly as expected in the last 6 years, despite the ‘test and treat’ rollout in 2016 [2] (**Figure 1**).

**Figure 1:**
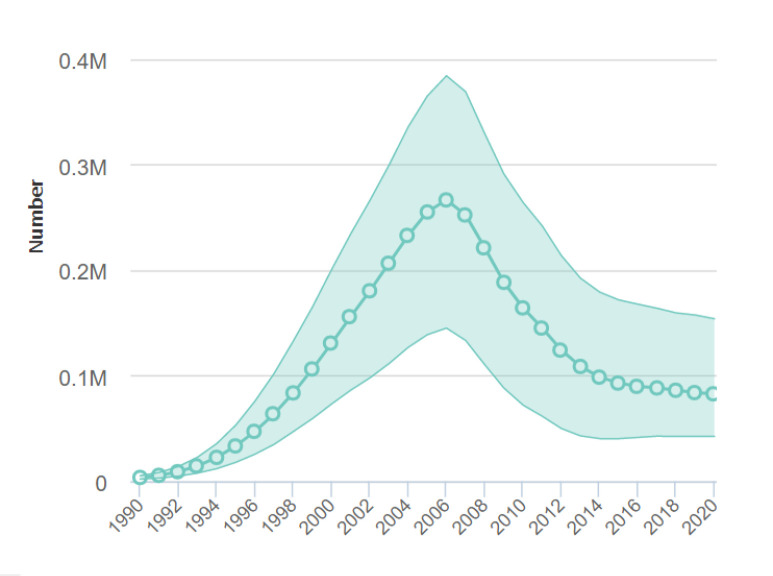
South Africa AIDS-related deaths (all ages) 1990-2020.

There is growing evidence of continued persistent advanced HIV disease (defined as CD4 < 200cells/mm^3^) from both late initial presentation which remains relatively unchanged over the last decade [4-7], and increasing disengagement from ART care [5,8,9]. This burden of advanced HIV disease accounts for considerable morbidity and mortality and high-cost hospitalizations [10-12] as well as high levels of drug resistance [13,14]. CD4 trends are similar from low to high income countries [15] and mortality is underreported [16]. Treatment as prevention of HIV (TasP) also requires that implementation challenges be met to achieve the desired population effect, which has not been seen as consistently as was optimistically anticipated [17,18].

CD4 count testing formed the backbone of historical HIV treatment programmes as a measure of immunocompromise, signifying the need to start ART, as well as for treatment monitoring in many countries. With the emerging evidence of the benefit of ART for all people living with HIV (PLWH), CD4 count thresholds were removed in December 2015 by the World Health Organization (WHO) [19], with universal test and treat (UTT) being adopted in South Africa in 2016. In 2017 the WHO released guidelines for advanced HIV disease, delineating a differentiated care package for those with low CD4 counts [20]. However, CD4 testing has been neglected in the UTT era with baseline CD4 counts missing in up to a third of cases [15,21,22]. The proportion of patients with advanced HIV disease in South Africa remained unchanged from 2012-2016 [6], concurring with the plateau in AIDS-related deaths. The importance of baseline and ongoing CD4 monitoring during advanced HIV disease has been emphasized across many settings [23-25].

Point of care (POC) CD4 testing has been shown to be accurate [26,27] (particularly at lower CD4 counts) and cost-effective [28,29], and has been used in many studies (both home and facility-based), that facilitated linkage to care, ART uptake [30-32] and viral suppression [33,34]. It has also been found to be acceptable [35] and requested by health care workers [36].

This study was conducted in a primary health care (PHC) clinic in Durban, South Africa, to evaluate if POC CD4 testing improved ART initiation, time to treatment initiation and linkage to care.

## METHODS

A time-randomized effectiveness-implementation study was conducted at Lancers Road PHC clinic, adjacent to a busy taxi rank in the Durban city-center from April to October 2015. The clinic initiated over 2500 patients on ART in 2015. HIV positive adults, ART naïve and not pregnant, requiring CD4 testing were enrolled. Weeks were randomized to either: standard-of-care (SOC) central laboratory CD4 testing sent from the clinic via the Addington Hospital Laboratory to the Prince Mshiyeni Hospital for flow-cytometry CD4 testing; or Alere ^TM^ PIMA POC CD4 (now Abbot PIMA^TM^ CD4) testing at the clinic. SOC weeks were 2; 4; 6-7; 11-14; 17; 20; 22; 24; 25 (13 weeks) and POC weeks were 1; 3; 5; 8-10; 15-16; 18-19; 21; 23; 26 (13 weeks). Participants were referred by clinic staff for testing and no participants refused participation. Voluntary written informed consent was obtained from all participants.

### Study intervention

3 PIMA analyzers were available for testing during PIMA randomized weeks and packed away on SOC weeks. Control beads were run each morning on the analyzers and all results were sent to Datapoint daily via the modem supplied by Alere. Study staff received Alere PIMA end user training with certificates. Study questionnaires were completed during the 20-minute PIMA specimen processing time during POC weeks. Participants were given the choice of fingerstick or venous blood draw (EDTA tube) for the POC testing with 148/299 (50%) choosing fingerstick. Participants known to be ART eligible were advised to have a venous draw to include required baseline bloods tests. All required phlebotomy was performed by the study staff. PIMA results were printed out (on supplied heat printers) and retained for study records. SOC participants were given a return date for 3-7 days to collect their results. Participants were integrated back into the clinic system and given a follow-up study visit appointment after 3 months if eligible for ART and 6 months if not yet eligible. These follow-ups were in-person or by telephone call if they did not attend a visit. Text message reminders were sent for follow-up visits if they did not attend. Study questionnaires collecting demographic and background information were administered at baseline and follow-up visits (Appendix S1 and S2 in the **Online Supplementary Document**). An exploratory objective included following-up the Lancers Road cohort until 5 years on ART from the clinic’s electronic ART records (part of the national Tier.net database).

### Statistical analysis

The study employed a cluster randomized design, where each week represents a cluster. Block randomization with block sizes of 4 were used to randomize across clusters. Assuming 60% of eligible patients in the SOC group initiate ART, sample sizes of 180 in each group (obtained by sampling 9 clusters of 20 subjects in each group) would achieve 80% power to detect a 15% improvement (ie, ART Initiation rate of 75% in the POC group). Block randomization with block sizes of 4 were used to randomize the POC/SOC across clusters. Generalised estimating equations models, with logit link were used to assess the relationship between study arm and starting ART within 90 days, while adjusting for correlation within cluster (randomization week). The probability of initiating treatment was presented graphically using Kaplan-Meier failure curves. Cox regression, with robust standard errors adjusting for clustering by week, were used to assess the relationship between treatment initiation and arm. Interaction effects between arm and CD4 count category were included in the models to conduct subgroup analyses. The Shapiro-Wilk test as well as quantile-quantile (Q-Q) plots and normal probability plots were used to assess normality assumptions of the continuous variables. Normally distributed variables were thus expressed as means with standard deviations, while continuous variables that violated normality assumptions were expressed as medians with interquartile ranges (IQRs). All analysis was conducted using Stata 15 (StataCorp, College Station, TX, USA).

### Ethics

Ethics approval was obtained from the Biomedical Research Ethics Committee of the University of KwaZulu-Natal (BF 480/14) and the eThekwini Research Ethics Committee. Clinical Trial Number: ISRCTN14220457.

## RESULTS

**Figure 2:**
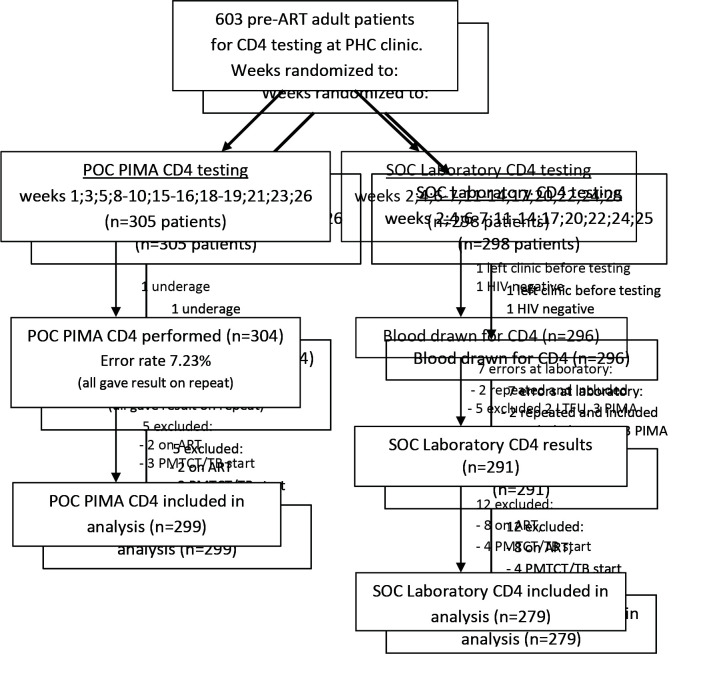
Flow diagram of cohort: HIV positive adults at Lancers Road PHC clinic, Durban (Not on ART; Not pregnant). SOC laboratory CD4 testing – standard-of-care central laboratory testing at the National Health Laboratory Services; POC PIMA CD4 testing – point-of-care PIMA^TM^CD4 testing at the clinic; PHC – primary health care; LTFU – lost to follow up; PMTCT/TB – prevention of mother to child transmission (pregnant) or tuberculosis ART start at any CD4 count

A flow diagram (**Figure 2**) details the randomization weeks and exclusions in each group.

### Baseline characteristics and history

There were no notable significant differences between the POC and SOC groups at baseline. **Table 1** shows the sociodemographic characteristics with 3 out of 4 patients being female. Almost half (47%) had not completed high school, a third (35%) had no form of employment and half (49%) were receiving state grants.

**Table 1:** Sociodemographic characteristics

Characteristic*	All	POC PIMA CD4 test	SOC laboratory CD4 test	*P*-value
Total n =	578	299	279	
Female	430 (74%)	221 (74%)	209 (75%)	0.731
Male	148 (26%)	78 (26%)	70 (25%)	
Age, median (IQR)	31 (26 - 37)	32 (25 - 38)	31 (26 - 37)	0.781
Female’s age, median (IQR)	31 (25 - 36)	31 (24 - 36)	31 (26 - 35)	
Male’s age, median (IQR)	33 (29 - 41)	33 (30 - 40)	34 (28 - 43)	
**Highest education:**				
None	8 (1%)	3 (1%)	5 (2%)	0.960
Less than secondary	268 (46%)	139 (47%)	129 (46%)	
Completed secondary	274 (47%)	144 (48%)	130 (47%)	
Tertiary	28 (5%)	13 (4%)	15 (5%)	
**Employment:**				
Full time formal	156 (27%)	78 (26%)	78 (28%)	0.326
Part time/casual/self employed	221 (38%)	129 (43%)	92 (33%)	
Unemployed	201 (35%)	92 (31%)	109 (39%)	
**Current partner:**				
Single	104 (18%)	54 (18%)	50 (18%)	
Has current partner	474 (82%)	245 (82%)	229 (82%)	0.962
Live with current partner (n = 469)	121 (26%)	66 (27%)	55 (24%)	0.433
Married (n = 470)	57 (12%)	29 (12%)	28 (13%)	0.664
Current multiple partners (n = 471)	52 (11%)	23 (9%)	29 (13%)	0.272
**Partner’s HIV status reported** (n = 469):	469	241	228	
Partner’s HIV status unknown	240 (51%)	126 (52%)	114 (50%)	0.801
Partner’s HIV status negative	54 (12%)	24 (10%)	30 (13%)	0.801
Partner HIV known infected	175 (37%)	91 (38%)	84 (37%)	
Partner, if infected on ART	91 (52%)	47 (52%)	44 (48%)	0.933
**Have biological children:**	455 (79%)	226 (76%)	229 (82%)	
No of biological children	2 (1 - 3)	2 (2-3)	2 (1 - 3)	0.325
Live with any of their children	148 (33%)	71 (31%)	77 (34%)	0.058
**Dwelling:**				
Formal	505 (87%)	252 (84%)	253 (91%)	0.022
Informal	73 (13%)	47 (16%)	26 (9%)	
**Income** (n = 576)**:**				
Receiving any welfare grants	284 (49%)	138 (46%)	146 (52%)	0.177
Monthly personal income - median ZAR	2000-3000	2000-3000	2000-3000	
US$	157-235	157-235	157-235	

POC – point-of-care, PIMA – Abbot PIMA^TM^CD4, SOC – standard of care, IQR – interquartile range, ART – antiretroviral therapy, ZAR – South African Rands, US$ - United States Dollars

*Denominators in brackets, depending on data available.

**Table 2** reveals baseline HIV and CD4 characteristics with a third (34%) of patients testing HIV positive for the first time. In patients with advanced HIV disease (CD4 ≤ 200cells/mm^3^), 40% tested HIV positive for the first time. The majority of those previously diagnosed were in the preceding year (41%), with almost a quarter knowing their status for more than 5 years. Over a third (36%) had never had a CD4 test despite knowing their HIV status and of those who had a previous CD4 count, 55% had been at a different health care facility.

**Table 2:** HIV status and prior CD4 testing

	TOTAL	POC PIMA CD4 test	SOC laboratory CD4 test	*P*-value
Total n-	578	299	279	
**HIV status**				
First HIV positive test on enrolment day	194 (34%)	103 (34%)	91 (33%)	0.641
Previously tested HIV positive	384 (66%)	196 (66%)	188 (67.4)	
Duration of knowing HIV positive status *n = 381:				
<6 months	105 (28%)	63 (32%)	42 (23%)	0.204
6-12 months	48 (13%)	26 (13%)	22 (12%)	
>1-3 years	96 (25%)	41 (21%)	55 (30%)	
>3-5 years	43 (11%)	20 (10%)	23 (12%)	
>5 years	89 (23%)	46 (24%)	43 (23%)	
Missing	3	0	3	
Months known HIV positive status, median (IQR)	14 (3-44)	12 (3-48)	18.5 (3-44)	0.347
Disclosed status previously	334 (87%)	171 (87%)	163 (87%)	0.874
**Previous CD4 count:**				
No previous CD4 despite prior positive HIV test	138 (36%)	76 (39%)	62 (33%)	0.237
Previous CD4 count taken – one or more	246 (64%)	120 (61%)	126 (67%)	
Knew previous CD4 test result (even estimated)	216 (82.4)	111 (88.9)	105 (76.4)	0.034
Didn’t know previous CD4 test result	30 (17.6)	9 (11.1)	21 (23.6)	
Previous CD4 at a different health facility n = 241	133 (55%)	67 (56%)	66 (54%)	0.731
Previous CD4 at this facility	108 (45%)	52 (44%)	56 (46%)	

POC – point-of-care, PIMA – Abbot PIMA^TM^CD4, SOC – standard of care, IQR – interquartile range

*Denominators depending on data available

### CD4 testing results

**Figure 3:**
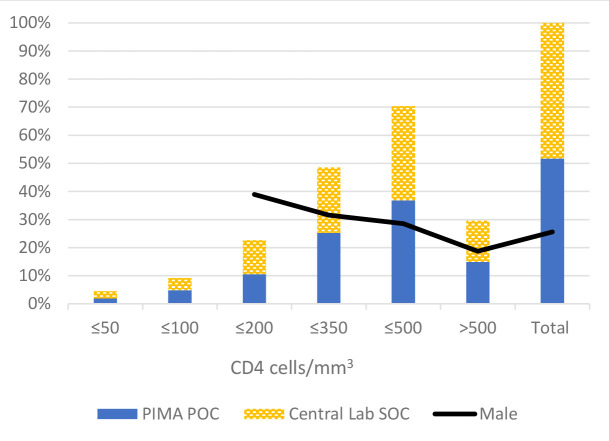
Cumulative CD4 counts for all participants per CD4 category n = 578. PIMA POC – Alere^TM^PIMA point of care CD4 testing,Central Laboratory SOC - Central Laboratory standard of care CD4 testing; Male – proportion of males within CD4 category

The CD4 results and distribution is seen in **Table 3**. The cumulative distribution by CD4 category is summarized in **Figure 3** with 407/578 (70%) of patients eligible for ART (CD4 ≤ 500 cells/mm^3^) while 131/578 (23%) had advanced HIV disease (CD4 ≤ 200 cells/mm^3^). There was no significant difference between the SOC and POC groups. Males had a lower median CD4 count of 256 cells/mm^3^ (IQR = 157-467) compared to females’ median CD4 383 cells/mm^3^ (IQR = 234-548). The overall proportion of males was 26% but increased in the ≤350 cells/mm^3^ category to 32% and in the ≤200 cells/mm^3^ category, with advanced HIV disease, males constituted 39%.

**Table 3:** CD4 count results

CD4 counts	Total n = 578	POC PIMA n = 299	SOC laboratory n = 279	*P*-value
Median (IQR)	357 (211-526)	357 (225-522)	356 (197-530)	0.808
≤50 cell/mm^3^	26 (5%)	11(4%)	15 (5%)	0.717
51-100 cell/mm^3^	27 (5%)	17 (6%)	10 (4%)	
101-200 cell/mm^3^	78 (13%)	33 (11%)	45 (16%)	
201-350 cell/mm^3^	150 (26%)	85 (28%)	65 (23%)	
351-500 cell/mm^3^	126 (22%)	67 (22%)	59 (21%)	
>500 cell/mm^3^	171(30%)	86 (29%)	85 (30%)	

POC – point-of-care, PIMA – Abbot PIMA^TM^CD4, SOC – standard of care, IQR – interquartile range

In the SOC group there were 7 laboratory errors, 2 of which were repeated, 2 were LTFU and 3 had PIMA (as returned in a PIMA week) and were therefore excluded. PIMA testing had an error rate of 7.3% with all giving a result on repeat testing.

### ART initiation and time to treat

**Figure 4:**
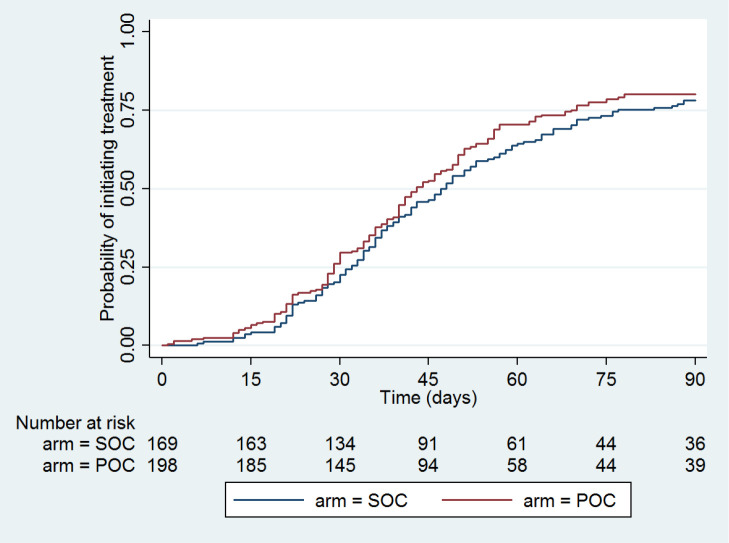
Kaplan-Meier curve of time to treat – OC PIMA vs SOC laboratory, all CD4 counts. SOC – standard-of-care central laboratory CD4 testing, POC – point-of-care PIMA^TM^ CD4 testing.

The primary end point analysis revealed that of all the participants eligible for ART (n = 406), 73% in the POC PIMA and 68% in the SOC Central laboratory group, initiated ART within 3 months/90 days, although this difference did not reach statistical significance. The time to treatment analysis excluded those eligible for ART who were LTFU after enrolment (n = 39) and revealed no significant difference across all patients eligible for ART at 3 months / 90days (HR = 1.12, 95% confidence interval (CI) = 0.85-1.48; *P* = 0.41) (**Figure 4**).

**Figure 5:**
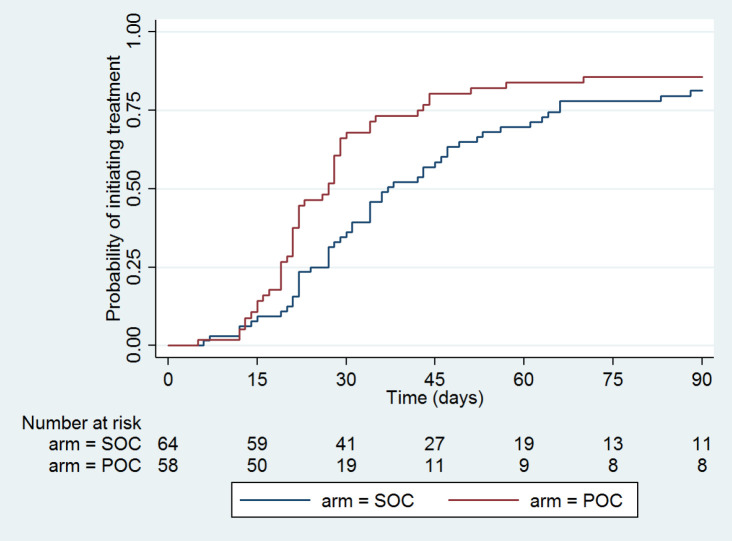
Kaplan-Meier curve of time to treat – POC PIMA vs SOC laboratory, advanced HIV disease (CD4 ≤ 200cells/mm^3^). SOC – standard-of-care laboratory CD4 testing, POC – point-of-care PIMA^TM^ CD4 testing.

When stratified by CD4 category (**Figure 5**), a difference between the POC and SOC groups was noted in the CD4 < 200 cells/mm^3^ category (HR = 1.65, 95% CI = 0.99-2.75; *P* = 0.052); effectively 65% more patients in the POC group started ART within 90 days. This however denotes only borderline significance due to the small sample number in this group. The supplementary material shows the other CD4 category’s Kaplan-Meier curves (Figure S1 in the **Online Supplementary Document**).

### Patient inconvenience

**Figure 6:**
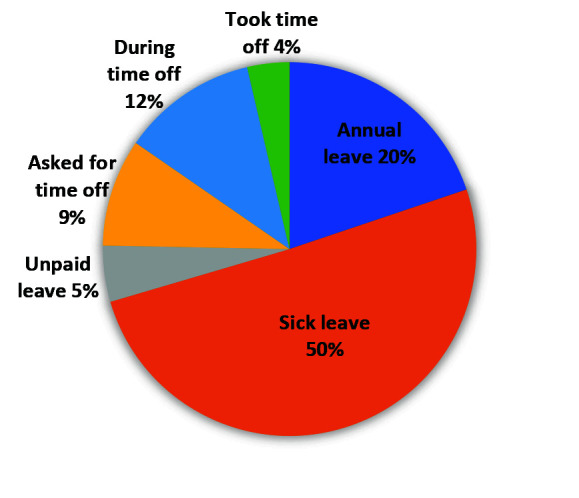
Leave types taken as reported by patients n = 248.

**Figure 6** depicts the types of leave taken as reported by 248/578 (43%) of patients. An additional 97(17%) students and learners may have missed school/lectures. A further 80/578 (14%) mentioned loss of income for coming to the clinic with a median of ZAR 100-150 (US$ 8-12) reported. The median transport cost was ZAR14 (US$1) (IQR = ZAR 10-21 (US$ 1-2)) per patient for the day for the 375/578 (65%) who paid for transport, while 31% walked to the clinic with a median travel time of 30-60 minutes per day for all participants.

### Retention in care

Retention in care did not differ significantly between the PIMA POC and SOC central testing groups. Of the cohort, 63/578(11%) were lost to follow up (LTFU) directly after their enrolment visit. Another 45/578 (8%) were found to have linked to care at other clinics upon follow up call: 24/45 (53%) within eThekweni, 8/45 (17%) in KwaZulu-Natal province, 7/45 (15%) in other provinces and 6/45 (13%) at unknown clinics and GPs. Of the patients who started ART at Lancers Road PHC, 17/410 (4%) never returned after their first ART initiation visit – and so it is unknown if they truly started treatment.

**Figure 7:**
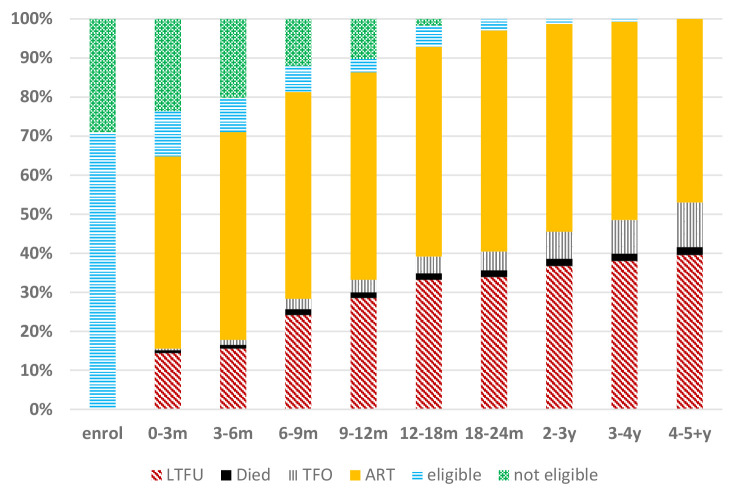
Lancers Road clinic 5-year ART retention n = 534 (2015–2020). LTFU – lost to follow up, TFO – transferred out, ART – on antiretroviral treatment, eligible – CD4 ≤ 500cells/mm^3^, not eligible – CD4 > 500cells/mm^3^.

**Figure 7** gives a summary of the Lancers Road cohort over 5 years. UTT was implemented in South Africa at the 12-18-month time point when all patients became eligible for ART. At 5 years, 251/534 (47%) were in care and 61/534 (11%) had transferred out to other facilities. Of the 211/534 (40%) who were LTFU at 5 years, 121/211 (57%) never started ART at Lancers Road PHC, which represented 121/534 (23%) of the total. Of the 323 patients who initiated ART at Lancers Road PHC, 15 (5%) re-engaged after being LTFU from ART over the 5yr period.

## DISCUSSION

This cohort tells the all too familiar story of HIV in Sub-Saharan Africa – of patients in and out of care, vulnerable patients, few men in care, poverty, and a heavily burdened clinic service [37]. Despite this, the cohort describes better ART initiation and linkage than many other services in South Africa which is a commendation to the dedicated staff at the clinic [6,38,39].

Previous CD4 testing had been at a different facility in 55% of patients presenting for care, 8% of the cohort linked to care at a different facility after testing, and 11% had transferred out from ART care by 5 years, highlighting the well documented mobility of patients accessing care [18,40,41].

The introduction of UTT in 2016 caused the ART initiation numbers to increase by a further 30%, with the patients with CD4 > 500 cells/mm^3^ becoming eligible and placing further strain on the health system.

UNAIDS proposed the 90-90-90 targets: 90% of PLWH know their status, 90% of those diagnosed with HIV receive ART and 90% of those on ART are virally suppressed. While the study was not designed to detect the 5% difference in time to treat, this difference is clinically meaningful as every patient on ART adds towards reaching the second ‘90’ target, which is currently at 72% [2] Previous studies have reported improvements in time to treat and linkage to care [31,42] while others have not [43-45].

The study design allowed a robust, time-randomized implementation method where all study participants were part of the same process, with half randomized to the POC intervention. This ensured that they all received the same time with a health care worker, counselling and information, which has been shown to be an important factor in ART initiation and linkage to care [46,47]. As patients still had to wait for baseline blood results from the central laboratory prior to initiation, same-day ART initiation could not occur in the POC group. Patients with CD4 counts above 200 cells/mm^3^ were also given appointments to return for ART initiation 4-6 weeks after eligibility due to high patient numbers, which interfered with the time to treat analysis for the groups – a real-world challenge in implementation studies [48].

Despite this cohort being prior to UTT rollout, it demonstrates that POC CD4 testing benefits those with advanced HIV disease significantly by identifying them and differentiating care for this vulnerable population. This benefit is even more applicable since UTT, where CD4 testing is being neglected and advanced HIV disease and mortality persists [4]. The higher proportion of men (40%) with advanced disease is not uncommon [38], and these men would likely have higher viral loads, thus transmitting the virus more readily. Engaging those who are continuing to drive the epidemic is a priority [49].

The five-year follow-up data for the cohort at Lancers Road clinic shows 47% of patients still in care at the clinic, with 11% having formally transferred to other clinics, and increasing LTFU with time. The deaths are likely underestimated and self-transfers are not able to be tracked beyond the follow-up calls. This is similar to other reports of retention in care at similar time periods [50,51]. This continuity of care requires a huge effort for a PHC clinic with 3723 patients in ART care in 2015 (now 8102 patients in ART care by November 2020) (Pers report Sr N Luthuli, Lancers Road Clinic). However, this retention and LTFU make the UNAIDS 90-90-90 targets [52] difficult to reach [53].

CD4 count is the primary method of assessment of advanced HIV disease, as even with a CD4 < 100cells/mm^3^, half of patients may have asymptomatic disease [54]. Advanced HIV disease was diagnosed in 23% of the cohort with 40% of these being diagnosed HIV-infected for the first time at enrolment. Prompt differentiation of care for these patients gives them the best chance of survival and recovery. This targeted management of advanced HIV disease is the WHO strategy to differentiate care to these vulnerable PLWH with the highest morbidity and mortality [20,55]. CD4 count is thus still essential for:

1. a baseline assessment for all at ART initiation,
2. continued monitoring for those with advanced HIV disease on prophylaxis regimens,
3. those presenting for ART after disengagement/ treatment interruption,
4. those with unsuppressed viral loads (due to poor adherence or treatment failure),
5. *ad hoc* CD4 testing for those presenting with symptomatic disease.

Point-of-care CD4 testing will allow health care workers to identify rapidly those requiring differentiated care for advanced disease, therefore providing the correct care, counselling, and education for this population at highest risk of morbidity, mortality, and ongoing transmission. Other valuable POC tests at this time point are creatinine and haemoglobin, the TBLAM urine test, Cryptococcal Ag test, Hepatitis B Ag test and a POC TB sputum test which would assist with a comprehensive diagnostic picture and ensure the comprehensive package of care is delivered, together with the adapted adherence support and follow-up as per WHO guidelines [20].

POC CD4 testing has been found to be acceptable to staff and patients [35] and requested by nurses [36]. A blood test is only valuable if it leads to better outcomes for the patient. POC testing assists with the same day actioning of a result by the health care team to ensure appropriate care, treatment, counselling, and follow-up for patients. This averts the delays experienced even in first world, highly computerised settings [56], which are a very common experience in our clinics with repeated testing and additional costs and time to the health system and to patients. POC testing can streamline services and allow more time for better care by decreasing the number of visits to the clinic and facilitating a ‘one stop shop’. Improving retention requires many more innovative and sensitive strategies to tackle the social, economic factors and unmet needs of PLWH who may cycle in and out of care over time [57-60].

A limitation of this study was that the POC testing was performed by study staff and not by the clinic staff, however other studies have shown feasibility and effectiveness of nurses, or even task shifting to lay health care workers, to perform PIMA testing [61]. The possibility of ‘silent transfers’ with patients moving to other clinics for care without official transfers when on ART or moving preART to other facilities could not be monitored. The 3–8-month follow-up calls to participants did document some of these ‘silent transfers”. However, this active follow-up of participants could also have been an added encouragement to patients and so increased their linkage to care beyond that of a non-research cohort giving an optimistic report of ART start and retention. Deaths were also likely to have been underestimated [16] as the national death register could not be accessed.

In the era of ‘test-and-treat’ the CD4 result is only reviewed after ART has started. This means that the vulnerable, low CD4 patients may be put at risk if they have TB or cryptococcal infection that may preferentially require treatment initiation before starting ART. POC testing for Cryptococcal Ag, TB (TBLAM and Xpert®MTB/RIF), Haemoglobin, Creatinine, Hepatitis B and HIV viral load are all commercially available[62]. Cotrimoxazole prophylaxis, for Pneumocystis pneumonia prevention, is also only initiated after ART start, and is commonly missed in as many 70% of patients [4]. This causes additional risk to the vulnerable patients and could be contributing to the plateauing of the HIV death rate in South Africa since 2012. Patients who default ART may return with low CD4 counts. In 2016 in the Western Cape, 52% of patients with CD4 < 50cells/mm^3^ had prior ART exposure [6]. They may not disclose their prior treatment and with the additional risk of drug resistance, they are at higher risk of dying [63]. Immunodeficiency associated with low CD4 counts also fuels the high TB incidence which compounds mortality in this vulnerable population [64].

In 2020, the WHO released guidance on the use of POC CD4 tests for advanced HIV disease with their product profile preferring a device-free test [65]. The benefit of a POC device that is connected is that data can be stored and reviewed centrally for reporting, monitoring and quality purposes and retrieved by health care workers at different clinics should patients move. This is in keeping with advancing mobile health or mHealth that is growing globally [66]. The PIMA^TM^CD4 devices are robust, tried and tested in the field, having WHO prequalification since 2012 and used widely in lower- and middle-income settings. With CD4 being linked to advanced HIV disease and thus mortality, it is important to monitor CD4s across maturing ART programmes to monitor these vulnerable patients. Next generation POC devices will perform multiple assays and combinations of assays within 10 minutes, which would further assist the clinical team in making on the spot decisions across the broad spectrum of communicable and non-communicable diseases for best patient care. Connectivity allows disease surveillance, quality monitoring and supply chain monitoring. South Africa has not implemented POC CD4 testing due to sufficient central laboratory capacity, but this deserves a clinical review.

## CONCLUSIONS

Where POC CD4 testing was previously found to improve assessment of ART eligibility and hasten ART start, it should now be used to improve rapid assessment of advanced HIV disease in those initiating ART, returning from interrupted ART and failing ART. This should include POC TBLAM testing (CD4 < 100-200 cells/mm^3^), Cryptococcal Ag testing (CD4 < 100 cells/mm^3^), and the differentiated care package required for these patients to improve care and decrease morbidity and mortality which will reduce the cost and health care burden of hospitalization. It is time to contend for those with low CD4 counts who are most likely to die of AIDS.

## Additional material


Online Supplementary Document
