# Supplementary material for: Point-of-care CD4 testing: Differentiated care for the most vulnerable
Source: J Glob Health. 2022 Feb 5;12:04004. doi: 10.7189/jogh.12.04004 (PMC8818294; doi:10.7189/jogh.12.04004)
Supplement: Online Supplementary Document [file jogh-12-04004-s001.pdf]

Appendix S1: PIMA II Questionnaire

Study no.______________ **PIMA** ☐ **NHLS** ☐ **Date:______________**

We would like to ask you some background questions to understand more about people with HIV, their circumstances and how they get care. All your information is confidential and the people reading the results will never know who you are.

1. Age ☐ ☐ years

2. Sex M ☐ F ☐

3. Tests

3.1 Did you know you were HIV positive before today? Yes ☐ No ☐

3.1.1 Can you tell me when and where you had your previous HIV tests? (don't have to be in order)

Date: DD/Mon/Yr	Result POS/NEG/IND (Indeterminate)	Where? 1= this clinic 2= another government facility 3= private doctor, hospital

If **No** to 3.1, go to 3.3.2

3.2 Have you disclosed your status to anyone? Yes ☐ No ☐ If **No** – go to 3.2.2

3.2.1 If **Yes**, who have you told? (can tick more than one)

Individual	Y/N	Date-mon,yr	Individual	Y/N	Date-mon,yr
a. Spouse or partner			d. Friend or non family member		

b. Relative in your house			e. Health care provider		
c. Relative not living with you			f. Other-specify……………………............		

3.2.2 If **No**, why haven't you disclosed to anyone? (Can tick more than one, don't give suggestions – wait for their responses)

a. Not ready		e. Fear of partner leaving	
b. Fear of stigma		f. It's my business	
c. They would judge me		g. I don't believe it	
d. Don't know		h. They wouldn't understand	

Other____

____

3.3 Have you had your CD4 count tested before? Yes ☐ No ☐

3.3.1 If **Yes**:-

Date DD/MON/YR	Result or +/- result or UNK	Where? 1=this facility, 2=other government facility, 3= private facility

3.3.2 What does the CD4 result mean to you?

____

____

____

____

(A CD4 count is a blood test that shows how much the HIV virus has affected your body and if it has weakened your body's defenses and killed the CD4 'soldiers')

4.<u>Partner</u>

4.1 Do you have a partner at the moment? Yes ☐ No ☐ (Partner 1) If **No**… go to 4.3

4.1.1 Do you know his/her HIV status?

	HIV status? POS, NEG,UNK	Last CD4 result?	On ART?	Last VL result?	Where? 1=this facility, 2=other government facility, 3= private facility
Partner 1					

4.1.2 Are you married? Yes ☐ No ☐ Widowed ☐ …if **Yes** legal ☐ or traditional ☐

4.2. Do you have any other partners at the moment?

Yes ☐ No ☐ Unwilling to disclose ☐ If **No** or '**Unwilling to disclose'** go to 4.3

4.2.1 If **Yes**, do you know their status?

(fill in on the below table if they are happy to talk about this)

	HIV Status? POS,NEG,UNK	Last CD4 result?	On ART?	Last VL result?	Where? 1=this facility, 2=other government facility, 3= private facility
Partner 2					
Partner 3					
Partner 4					

4.3 Do you know if any of your previous partners were HIV positive?

Yes ☐ No ☐ (if **Yes**…. fill in the table below)

Previous Partner	Year	Last CD4?	On ART?	Last VL?	Where? 1=this facility, 2=other government facility, 3= private facility
Partner 1					
Partner 2					
Partner 3					
Partner 4					

5. <u>Education</u>

a. None		e. High School - completes	

b. Primary – did not complete		f. Certificate study – eg. S1,N1 etc.	
c. Primary – completed		g. Tertiary / After school	
d. High School – did not complete			

6. Household

6.1 Could you indicate the type of dwelling that you occupy at the moment?

a. Dwelling/House or brick structure on a separate stand or yard or on farm	A
b. Traditional dwelling/ Hut/ Structure made of traditional material	B
c. Flat or apartment in a block of flats	C
d. Town house /semi-detached house in a complex	D
e. Semi detached house	E
f. House/Flat/room in backyard or in a house	F
g. Informal dwelling/Shack in backyard	G
h. Informal dwelling/Shack not in backyard, e.g. in an informal/squatter settlement or on farm	H
i. Caravan/Tent	I
j. Other, specify………………………………………………………………..	J

6.2 Please tell me which of the following, if any, are presently in your household (in working order). Does your household have…?

ITEM	Y / N
a. Cell phone/ Mobile phone	
b. A car that is working	
c. Hot running water	
d. Fridge/freezer combination	
e. Microwave oven	
f. Electric stove	
g. Television	

h. DVD player in household	
i. Washing machine	
j. Vacuum cleaner	
k. Telkom home phone	
l. Computer at home	
m. Hi-fi or music centre	
n. Built-in kitchen sink	
o. A deep freezer	

6.3 How many live in your household? (excluding you)

Adults :- ☐ ☐ Children : ☐ ☐

6.4 Do you know the HIV status of the other adults in your household?

No.	Relationship	Status? Pos,Neg or UKN	On ARV? Y, N, UNK or N/A	Where? 1=this facility, 2=other government facility, 3= private facility
1				
2				
3				
4				
5				
6				
7				

6.5 Do you have children that you gave birth to, or are the father of? Yes ☐ No ☐

6.5.1 If **Yes**, please complete the table?

Child No.	Age	Stay with you? Y / N	HIV Status? Pos, Neg, UNK	On ART? Y, N, UNK	Where? 1=this facility, 2=other government facility, 3= private facility
1					
2					
3					
4					

5					
6					

6.6 Are there other children in your household? Yes ☐ No ☐

6.6.1If **Yes** please complete the table below.

Child No.	Age	HIV Status? POS, NEG, UNK	On ART? Y, N, UNK	Where? 1=this facility, 2=other government facility, 3= private facility
1				
2				
3				
4				
5				
6				

7. <u>Food Security</u>

7.1 In the past [4 weeks/30 days], was there ever no food to eat of

any kind in your house because of lack of resources to get food?

Yes ☐ No ☐ (if no go to 7.2)

7.1.1 How often did this happen in the past [4 weeks/30 days]? (**Circle** the number)

1 = Rarely (1–2 times) 2 = Sometimes (3–10 times) 3 = Often (more than 10 times)

7.2 In the past [4 weeks/30 days], did you or any household member go to sleep at night hungry because there was not enough food?

Yes ☐ No ☐ (if no go to 7.3)

7.2.1 How often did this happen in the past [4 weeks/30 days]? (**Circle** the number)

1 = Rarely (1–2 times) 2 = Sometimes (3–10 times) 3 = Often (more than10 times)

7.3 In the past [4 weeks/30 days], did you or any household member

go a whole day and night without eating anything at all because there was not enough food?

Yes ☐ No ☐ (if no go to 8)

7.3.1 How often did this happen in the past [4 weeks/30 days]? (**Circle** the number)

1 = Rarely (1–2 times) 2 = Sometimes (3–10 times) 3 = Often (more than 10 times)

8. Employment

Which of the following best describes your present work situation?

a. Employed full time	A
b. Employed part time	B
c. Employed less than part time (casual work/piecework)	C
d. Temporarily sick	D
e. Unemployed, not looking for work	E
f. Unemployed, looking for work	F
g. Housewife, not working at all, not looking for work	G
h. Housewife, looking for work	H
i. Student/learner	I
j. Pensioner (aged/retired)	J
k. Permanently sick or disabled	K
l. Volunteering	L
m. Other (specify) …………………………………………………………………	M

9. Income

9.1 Do you or anyone in your household receive any of the following welfare grants?

GRANT	YES	NO	Amount?
a. Old Age Grant			
b. Child Support Grant			

c. Disability Grant			
d. Care dependency grant			
e. Foster care grant			
f. Grant in aid			
g. Don't know the name of grant			
h. No-one in household receiving any benefits			
**GRANT**	**YES**	**NO**	**Amount?**
i. (Did not want to answer)			
j. (Don't know)			

9.2 Please CIRCLE the letter that best describes the TOTAL MONTHLY HOUSEHOLD INCOME of all the people in your household before tax and deductions if you know.(including grants)

HOUSEHOLD INCOME **or write down amounts**

No income	A	
<R1000	B	
R1001 – R1500	C	
R1 501 – R2 000	D	
R2 001 – R3 000	E	
R3 001 – R5 000	F	
R5 001 – R7 500	G	
R7 501 – R10 000	H	
R10 001 – R15 000	I	
R15 001 – R20 000	J	
R20 001 – R30 00	K	
R30 001 – R50 000	L	
R 50 001 +	M	
(Did not want to answer)	N	
(Uncertain/Don't know)	O	

9.3 Please circle the letter that best describes your PERSONAL TOTAL MONTHLY INCOME before tax and other deductions.

PERSONAL INCOME

No income	A
R1 – R500	B
R501 –R750	C
R751 – R1 000	D
R1 001-R1 500	E
R1 501 – R2 000	F
R2 001 – R3 000	G
R3 001 – R5 000	H
R5 001 – R7 500	I
R7 501 – R10 000	J
R10 001 – R15 000	K
R15 001 – R20 000	L
R20 001 – R30 00	M
R30 001 – R50 000	N
R 50 001 +	O
(Did not want to answer)	P
(Uncertain/Don’t know)	Q

10. <u>Leave</u> if patient is not working go to 11.

10.1 Did you need to take leave for today?

Yes ☐ No ☐

10.1.1 If **Yes**, was your leave

	YES	NO
a. Unpaid leave		
b. Sick leave		

c. Annual leave		
d. Family Responsibility leave		
e. Other…………………..		

10.2 Can you tell me your loss of earnings coming to the clinic if any?

None ☐ R1-R50 ☐ R50-R100 ☐ R100-R150 ☐

R150-R200 ☐ >R200 ☐

11. <u>Transport</u>

11.1 How did you get to the clinic?

Walk ☐ Lift ☐ Own Car ☐ Bus ☐ Taxi ☐ Train ☐

Other____________________________

11.2 How long did it take you to get here?

< 15 min ☐ 15-30 min ☐ 31-45 min ☐ 46-60 min ☐

61-90 min ☐ 91-120 min ☐ >120 min ☐

(1- 1.5 hours) (1.5-2 hours) (more than 2 hours)

11.3 Did you come to the clinic with someone? Yes ☐ No ☐ If **No** go to 11.6

11.3.1 If yes - who did you come with? _______________________________

11.3.2 Please tell me about this person/people and why did they come with you:

(**Probe** – were their medical reasons for this person to come? If a child – age?)

____

____

____

____

____

11.4 Total cost for all your transport coming to the clinic? R____________

11.5 Total cost for you all to get back? R____________

11.6 How much did it cost you alone to get here? R____________

11.7 How much will it cost you alone to get back? R____________

11.8 Where do you go after leaving the clinic?

Work ☐ Home ☐ Shopping ☐ to a Friend ☐

Other____

THANK YOU FOR YOUR TIME

Time__________ **RA Sign______________________________**

____

CD4 RESULT

CD4 _________ Time____________ Date________________

12. What was your response to your CD4 count?

____

____

____

____

13. Would you have preferred to have a fingerstick or venous sample taken for CD4 count?

Fingerstick ☐ Venous draw ☐ Don't mind either ☐

14. Eligible for ART? Yes ☐ No ☐ (CD4<500, TB patient, Pregnancy, HepB…)

15. Baseline bloods taken Yes ☐ No ☐ Date______________

THANK YOU RA Sign_______________ Date____________

Appendix S2: PIMA II Follow–on Questionnaire

Study no.________________ Face to face ☐ Phone ☐ Date:_________________

We would like to ask you some follow up questions to understand about more about what has happened to you since we last chatted. All the answers you give are confidential and the people reading them will never know who you are. Some questions are the same – we are just confirming or things might have changed.

1. Age ☐☐

2. Sex **M** ☐ **F** ☐

3. Tests

3.1. Have you disclosed your status to anyone new since your visit here?

Yes ☐ No ☐ If **No** go to 3.1.3

3.1.2. If **Yes**, who all have you told?

Individual	Y/N	Date-mon,yr	Individual	Y/N	Date-mon,yr
Spouse or partner			Friend or non family member		
Relative in your house			Health care provider		
Relative not living with you			Other-specify…………………….............		

3.1.3 If **No**, why haven't you disclosed to anyone? (Can tick more than one, don't give suggestions – wait for their responses)

Have disclosed already	A		
Not ready	B	Fear of partner leaving	F
Fear of stigma	C	It's my business	G
They would judge me	D	I don't believe it	H
Don't know	E	They wouldn't understand	I

Other____

____

____

3.2 Have you had your CD4 count tested again since last visit?

Yes ☐ No ☐

3.2.1 If **Yes**:-

Date DD/MON/YR	Result or UNK	Where? 1=this facility, 2=other government facility, 3= private facility

4.Partner

4.1 Has anything changed with your partner since we last spoke? Yes ☐ No ☐

If **No**.. go to question 5.

4.2 Do you have a partner at the moment? Yes ☐ No ☐ (Partner 1) If **No…** go to 5.

4.2.1 Do you know this partner's HIV status?

	HIV status? POS, NEG,UNK	Last CD4 result?	On ART?	Last VL result?	Where? 1=this facility, 2=other government

					facility, 3= private facility
Partner 1					

4.2.2 Are you married? Yes ☐ No ☐… if **Yes** legal ☐ or traditional ☐

4.3. Do you have any other partners at the moment?

Yes ☐ No ☐ Unwilling to disclose ☐ If **No** or '**Unwilling to disclose'** go to 5.

4.3.1 If yes, do you know their status? (fill in on the below table if they are happy to talk about this)

	HIV Status? POS,NEG,UNK	Last CD4 result?	On ART?	Last VL result?	Where? 1=this facility, 2=other government facility, 3= private facility
Partner 2					
Partner 3					
Partner 4					

5. <u>Household</u> – has anything changed in your household since we last spoke? Scan through the questions to check if necessary. If **No** – go to **6.**

5.1 How many live in your household? (excluding you)

Adults :- ☐☐ Children :- ☐☐

5.2 Do you know the status of the other adults in your household? (patient is Adult no. 1)

Adult no.	Relationship	HIV Status? Pos, Neg or UKN	On ART? Y, N, UNK, N/A	Which clinic? Name, UNK or N/A
2.				
3.				
4.				
5.				
6.				

5.3 Do you have new children that you gave birth to or are the father of? Yes ☐ No ☐

5.3.1 If **Yes**, please complete table below:-

Child No.	Age	Stay with you? Y N	HIV Status? Pos, Neg, UNK	If POS- on ART? Y, N, UNK	Where? 1=this facility, 2=other government facility, 3= private facility
1.					
2.					
3.					
4.					
5.					
6.					

5.4 Are there other children in your household? Yes ☐ No ☐

5.4.1If **Yes** please complete the table below:-

Child No.	Age	HIV Status? POS NEG UNK	If POS - on ART? Y, N, UNK	Where? 1=this facility, 2=other government facility, 3= private facility
1.				
2.				
3.				
4.				
5.				
6.				

6. <u>Retention</u>

6.1 Were you given your CD4 result at your last visit? Yes ☐ No ☐

if **Yes** go to 6.3 If **No** :-

6.2 Why did you not receive it? (don’t prompt reply, can tick more than one response)

Too busy to return	A	Result lost	C
Didn’t want to know result	B	Too afraid to find out	D

Other____

____

____

6.3 Did you get your result?

Same day ☐ Later date ☐

6.3.1 When would you have preferred to get your results?

Same day ☐ Later date ☐ Didn't mind ☐

6.4 Do you remember your CD4? Yes ☐ No ☐ If **No** remind them.

CD4 __________ Date__________________

6.5 Are you eligible for ARVs? Yes ☐ No ☐ Don't know ☐

(if they don't know tell them if they are eligible, explain and go to end.) If **Yes** – go to 6.5.2

6.5.1 If **No** – when is your next CD4 due?

6 months –correct answer	A	Comment :
Incorrect answer – any other time frame	B	
Don't know	C	

(Inform them to come back in 6 months if didn't know)

6.5.2 If **Yes** – have you started ARVs? Yes ☐ No ☐

If **No** go to 6.5.3

6.5.2.1 If **Yes**..... Date started _______________

6.5.2.2 At which clinic? _________________________________

6.5.2.3 What is your ARV file no?___________ (UNK if don't know)

6.5.3 If **No** – can you give me the reason(s) why?

Can tick more than one reason. Don't give any prompts from the list. Wait for them to say and write under other if not exactly as in the list.

**Personal factors**		**Clinic factors**	
I didn't know I needed them	A	On TB treatment	J
I'm feeling well	B	Waiting for results	K
I'm not ready (probe why?)	C	The clinic is too busy	L
I'm too busy	D	The queues are too long	M
I can't get off work	E	The staff are rude to me	N
I'm afraid of the drug side effects	F	I relocated and haven't been to another clinic	P
I don't want to (probe why?)	G	I was sent to another clinic and have not been	Q
I will start in …………..	H	I wait too long at the clinic	R
Transport costs too much	I		S

Other____

____

____

____

____

THANK YOU **RA name**_______________________ **RA sign**____________________

Figures S1

Figure 8. CD4 counts 201-350 cells/mm^3: SOC = Standard of care central laboratory CD4 testing ; POC = Point of care PIMA™CD4 testing

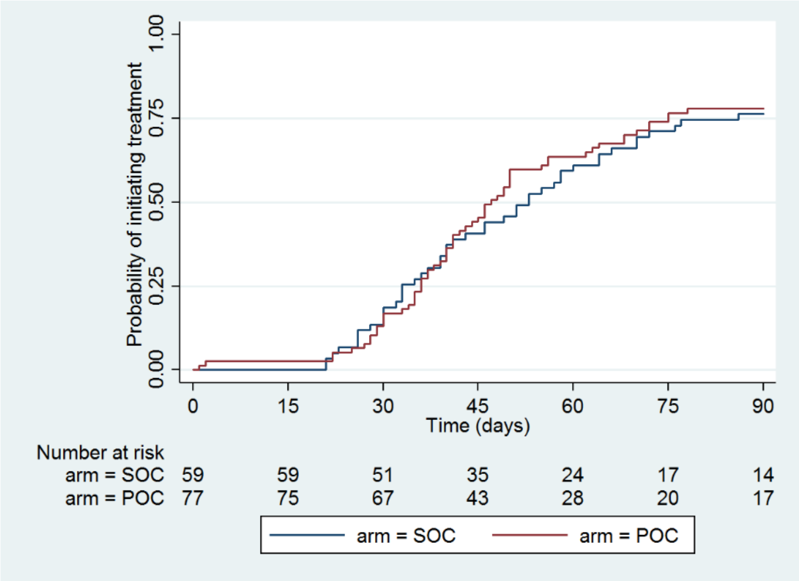

HR 1.07 (CI: 0.67 – 1.73; p = 0.77)

Figure 9. CD4 counts 351-500 cells/mm^3: SOC = Standard of care central laboratory CD4 testing ; POC = Point of care PIMA^{TM}CD4 testing

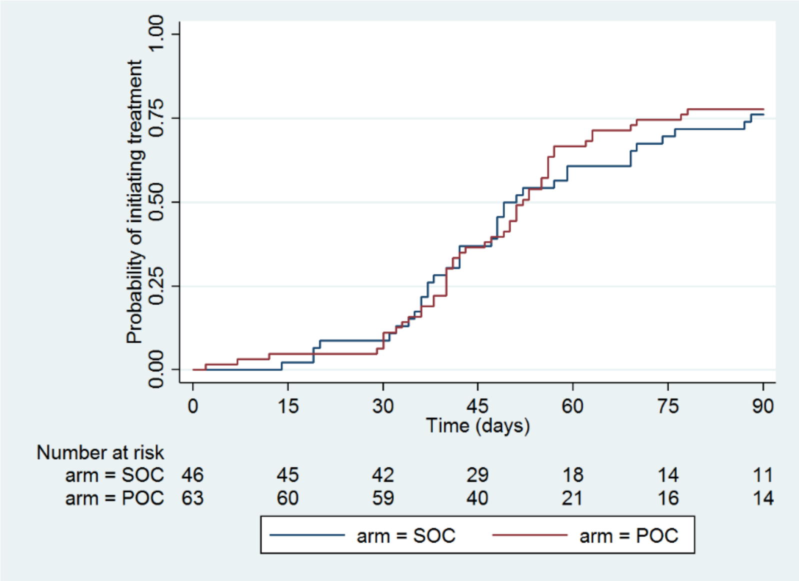

HR 1.04 (CI: 0.72-1.52; p = 0.83)
